# Novel Approaches to Improve Myeloma Cell Killing by Monoclonal Antibodies

**DOI:** 10.3390/jcm9092864

**Published:** 2020-09-04

**Authors:** Paola Storti, Federica Costa, Valentina Marchica, Jessica Burroughs-Garcia, Benedetta dalla Palma, Denise Toscani, Rosa Alba Eufemiese, Nicola Giuliani

**Affiliations:** 1 Department of Medicine and Surgery, University of Parma, 43126 Parma, Italy; federica.costa@unipr.it (F.C.); valentina.marchica@unipr.it (V.M.); jib6x6@gmail.com (J.B.-G.); benedetta.dallapalma@gmail.com (B.d.P.); denise.toscani@unipr.it (D.T.); eufemieserosalba@gmail.com (R.A.E.); nicola.giuliani@unipr.it (N.G.); 2 Department of Medical-Veterinary Science, University of Parma, 43126 Parma, Italy

**Keywords:** monoclonal antibody, multiple myeloma therapeutic targets, CD38, SLAMF7

## Abstract

The monoclonal antibodies (mAbs) have significantly changed the treatment of multiple myeloma (MM) patients. However, despite their introduction, MM remains an incurable disease. The mAbs currently used for MM treatment were developed with different mechanisms of action able to target antigens, such as cluster of differentiation 38 (CD38) and SLAM family member 7 (SLAMF7) expressed by both, MM cells and the immune microenvironment cells. In this review, we focused on the mechanisms of action of the main mAbs approved for the therapy of MM, and on the possible novel approaches to improve MM cell killing by mAbs. Actually, the combination of anti-CD38 or anti-SLAMF7 mAbs with the immunomodulatory drugs significantly improved the clinical effect in MM patients. On the other hand, pre-clinical evidence indicates that different approaches may increase the efficacy of mAbs. The use of trans-retinoic acid, the cyclophosphamide or the combination of anti-CD47 and anti-CD137 mAbs have given the rationale to design these types of combinations therapies in MM patients in the future. In conclusion, a better understanding of the mechanism of action of the mAbs will allow us to develop novel therapeutic approaches to improve their response rate and to overcome their resistance in MM patients.

## 1. Introduction

In recent years, the introduction of monoclonal antibodies (mAbs) targeting CD38 and the signaling lymphocytic activation molecule family member 7 (SLAMF7) represents an important step towards the treatment of relapsed/refractory multiple myeloma (RRMM) patients [1,2,3].

More recently, the use of mAbs is moving into the first line treatment of newly diagnosed MM patients with high rate and durable responses [2,4,5].

Although immunotherapy with mAbs represents an attractive approach because of its well-established clinical efficacy, there is substantial variability in the sensitivity and duration of the response among patients. In this review, we will specifically focus on the mAbs currently used in the treatment of MM, such as the anti-CD38 antibodies daratumumab (DARA), isatuximab (ISA) and the anti- SLAMF7 elotuzumab (ELO). We will provide a summary of their mechanisms of actions and the new strategies to improve their effectiveness and overcome resistance.

## 2. Mechanisms of Action

### 2.1. Anti-CD38 Monoclonal Antibodies

DARA is the first CD38-targeting mAb approved in MM therapy. It is a fully human immunoglobulin G1 kappa (IgG1κ mAb that targets CD38 [6]. More recently, other anti-CD38 mAbs have been developed: ISA, an IgG1-κ chimeric mAb and MOR202, an IgG1-λ fully human mAb [7].

Anti-CD38 antibodies kill myeloma cells by different mechanisms of action (MoA), including classical FC-dependent immune effector mechanisms, direct and immunomodulatory effects [8]. Anti-CD38 antibodies can bind the Fc gamma receptors (FcγRs) on the immune effector cells inducing the antibody-dependent cell-mediated cytotoxicity (ADCC) and antibody-dependent cellular phagocytosis (ADCP) [7]. Natural killer (NK) cells are the main mediator of ADCC by DARA, MOR202 and ISA. Also, CD14^+^CD16^+^ monocytes have a role in this mechanism of MM cell killing by DARA [8,9]. Moreover, phagocytosis contributes to the anti-MM activity of the anti-CD38 mAbs [8]. In vitro studies have demonstrated that DARA-coated MM cells are rapidly engulfed by macrophages [10]. Recently, it has been demonstrated that, in particular, the CD16^+^ (FcγRIIIA) subset of monocytes is fundamental in DARA MM cells-killing activity [11]. In vitro studies have demonstrated that MOR202 can induce ADCP by myeloma-associated macrophages against MM cell lines [12]. On the other hand, ISA triggers ADCP only on MM cells that present a high level of CD38 molecules on the surface [13].

Moreover, the Fc tail of the anti-CD38 mAbs can activate the complement cascade inducing the complement-dependent cytotoxicity (CDC) against MM cells [7]. DARA is the most effective inducer of CDC, while ISA can induce CDC only in a few MM samples with high expression of CD38 on plasma cells (PCs) [13].

DARA also has an immunomodulatory effect in the MM bone marrow (BM) microenvironment, depleting T regulatory cells (T regs), regulatory B cells (B regs), and myeloid-derived suppressors cells (MDSCs) [7,14,15]. As a result of the reduction of immuno-suppressor cells, DARA induces CD4^+^ and CD8^+^ T cells expansion in MM patients and in particular the effector memory CD8^+^ T cells concomitant with a decrease of naïve T cells subset [15]. Similar to DARA, ISA reduces T regs and blocks the production of immune inhibitory cytokines like interleukin (IL)-10 [16]. Moreover, CD38 is an ectoenzyme involved in the metabolism of nicotinamide adenine dinucleotide (NAD+) and adenosine: NAD+ reduction leads to the development of exhausted T cells and adenosine has an immunosuppressive effect on NK and CD8^+^ cells [17,18]. Indeed, targeting CD38 with anti-CD38 mAbs could restore the immune functions.

Finally, ISA directly induces MM cell death by binding the CD38 on the cell surface, then activating the classical caspase and lysosome death pathways [19]. In contrast, neither DARA nor MOR202 showed a direct killing effect on MM cells.

The tissue expression profile of CD38 explains some of the “off target” effects of DARA. It has been reported that CD38 is also expressed by red blood cells (RBCs). Binding of DARA to CD38 on RBCs leads to panagglutination in indirect antiglobulin test, possibly masking clinically relevant alloantibodies and complicating the selection of compatible RBCs for transfusion [20]. On the other hand, in both pre-clinical studies and clinical trials, it has been reported that DARA does not induce clinically relevant hemolysis, probably, either due to the low CD38 expression levels on RBCs or the capacity of DARA to induce antigen loss on the surface of RBCs [21].

The use of therapeutic mAbs may also affect laboratory diagnostics in MM patients. It has been reported that DARA interferes with PCs detection by flow cytometry [22] and disturbs the detection and quantitation of M-protein by immunofixation electrophoresis (IFE) [23].

Clinical studies showed that DARA can be detected as an individual monoclonal band in serum IFE. Because M-protein follow-up by IFE is part of the criteria to assess the response to the treatment in MM patients, therefore, DARA band must not be confused with the endogenous M-protein of the patient. To discriminate between endogenous M-protein and DARA, a DARA-specific immunofixation electrophoresis reflex assay (DIRA) was developed using a mouse anti-DARA antibody [24]. Similarly, it has been reported the interference with anti-CD38 antibody for PC detection by flow cytometry that persist from four months to six months after treatment with daratumumab. Alternative mAbs may be used. VS38 antibody anti-CD38 may be used for the detection of PCs after treatment [25]. Clearly the possible interference with laboratory testing can be also seen with the other anti-CD38 therapeutic antibodies.

### 2.2. Anti-SLAMF7 Monoclonal Antibody

ELO is a humanized IgG1 mAb that targets the extracellular domain of SLAMF7, which is expressed on normal PCs and MM cells [26]. SLAMF7 is expressed at a lower level on NK cells, CD8+ T cells, mature dendritic cells and activated T cells [26]. The primary mechanism of action of ELO is via NK-ADCC. ELO-induced ADCC is mediated through the binding of its Fc portion with CD16 on NK cells [27]. Studies performed by Hsi et al. showed that ELO could bind SLAMF7 present on the surface of myeloma cells and directly induce the activation of NK cells via Fc receptors. The activation of NK cells triggers the release of perforin granules that target MM cells, enhancing the anti-tumor effect of ELO [27]. In a mouse model, it has also been demonstrated that ELO can induce ADCP by M1 polarized tumor associated macrophages against human MM cell lines [28].

## 3. Mechanisms of Resistance

The mechanisms involved in the acquired resistance to therapy with mAbs are not fully understood. Downregulation of the target antigen may be one of the factors that contribute to the development of mAbs resistance. However, in clinical settings, the correlation between CD38 expression before treatment and responses to DARA therapy is still controversial [29,30]. Studies carried by Nijhof et al. demonstrated that MM cell lines expressing higher levels of CD38 were more susceptible to DARA-mediated ADCC and CDC, compared to those cell lines with lower expression of CD38 [31]. Recently, the same group demonstrated an association between CD38 expression levels and DARA efficacy to induce cell death by ADCC and CDC. Specifically, Nijhof and colleagues showed that MM patients who achieve partial remission when treated with DARA had a higher CD38 expression level than patients who did not achieve partial remission [32]. These results suggest that CD38 expression levels before treatment can be a key determinant to achieve a positive outcome when patients are treated with this drug. On the other hand, two studies reported no differences in the levels of CD38 expression between responders and non-responders prior to the administration of DARA [29,30].

Danhof et al. reported that other factors, like an increased number of NK cells prior to the treatment, could potentiate the patient′s response when treated with ELO [33]. Additionally, polymorphisms in FcγRIIIa V allele (CD16a) might also be important for ELO efficacy, although these results are not conclusive [33]. The FcγRIIIa gene contains allelic variations that confer the affinity of FcγRIIIa to the mAb [27]. In a randomized phase II study of ELO and Dexamethasone, Jakubowiak and colleagues reported that patients homozygous for the high affinity allele FcγRIIIa V showed a longer progression-free survival (PFS) than those who were homozygous for the low-affinity allele [33]. However, FcR polymorphisms have only a modest impact on the response to DARA and did not significantly affect the overall survival (OS) [34].

In addition, the modulation of CD38 after treatment with a mAb has also been studied as a possible mechanism of acquired resistance. DARA induces the release of CD38 from myeloma cells by microvesicles [35] and the CD38 expression on MM cells is decreased after DARA exposure due to the process called trogocytosis, where there is the transfer of CD38 of MM cells to monocytes and granulocytes [36]. On the other hand, in vitro studies showed that treatment with ISA does not decrease the surface expression of CD38 on MM cells [13].

In addition, DARA resistance was found to be associated with the overexpression of complement inhibitory proteins CD55 and CD59 at the time of disease progression [32].

## 4. Improving the Efficacy of mAbs and Overcoming Resistance

### 4.1. All-Trans Retinoic Acid (ATRA)

The expression of targets on the surface of MM cells appears to be the main determinant for the resistance to mAbs. The modulation of the expression of these targets by different agents that can re-sensitize mAbs seems to be a possible key to overcoming resistance to the drug. In vitro and in vivo studies showed that agents such ATRA can be used to overcome resistance by increasing the expression of CD38 in MM cells [31]. Nijhof et al. investigated the role of ATRA in cell lines and primary MM cells [31]. Specifically, they showed that treatment with ATRA significantly increased the expression of CD38 enhancing DARA-induced ADCC and CDC in vitro. Likewise, in a humanized mouse model, ATRA improved the anti-MM effect of DARA [31]. The mechanism involving the modulation of CD38 by ATRA can be explained by the presence of a retinoic acid responsive element located in the first intron of the CD38 gene [37]. Interestingly, treatment with ATRA also reduced the expression of CD55 and CD59 in MM cells isolated from both DARA-naïve patients and patients with DARA-refractory disease [32]. These studies provided the rationale for the ongoing clinical trial with ATRA and DARA to treat DARA-naïve RRMM patients. Further studies combining CD38 mAbs with ATRA are needed to potentiate and recapture mAbs responses.

### 4.2. Anti-CD47 Monoclonal Antibody

Among the new potential therapeutic mAbs, CD47 is emerging as a new target in the context of solid tumor and hematological malignancies. Here, we reported the potential role of targeting CD47 as a therapeutic option alone or in combination with the main drugs used in MM treatment, to explain emerging immunotherapeutic approaches.

CD47 is an integrin-associated receptor, ubiquitously expressed on the surface of many cell types including RBCs and cancer cells [38,39,40]. The principal role of CD47 is to inhibit the phagocytosis through the binding of the signal regulatory protein-α (SIRPα) [41]. In particular, CD47 expressed on target cells interacts with SIRPα expressed on phagocytic cells, thus activating a “don’t eat me” signal [42]. The inhibition of this axis promotes the killing of tumor cells by macrophages and neutrophils [43]. The CD47/SIRPα axis was defined as an innate immune checkpoint in several tumor models, which stimulates antigen-presenting cell function and consequently the adaptive T cell-mediated anti-tumor immunity [44,45]. Several studies have reported the use of anti-CD47 in hematologic malignancies with promising results as anti-tumor therapy alone or in association with drugs used in clinical practice, including MM [46,47,48,49]. Indeed, it has been reported that the use of an anti-CD47 monoclonal antibody induces phagocytosis and killing of MM cells [50,51]. Several authors reported high expression levels of CD47 on different MM cell lines and PCs obtained from MM patients [50,52]. In particular, Sun et al. confirmed that CD47 was expressed ubiquitously in several BM sub-populations. Notably, PCs represent the population with the highest expression level of CD47. Moreover, the expression of CD47 increases with advanced stage of disease, from monoclonal gammopathy of undetermined significance (MGUS) to MM [52]. Sun et al. found that anti-CD47 antibody treatment of MM cells, co-cultured with macrophages, enhanced MM-killing, suggesting the involvement of macrophages in anti-CD47-mediated phagocytosis of myeloma cells [52].

Kim et al. reported that anti-CD47 antibodies require the involvement of phagocytic cells or their progenitors such as monocytes, macrophages, and dendritic cells to perform the anti-MM activity. In particular, they observed that the treatment with anti-CD47 mAb induces phagocytosis of MM cells by macrophages derived from mouse models. In addition, they showed that the anti-CD47 treatment influences the viability of cells; however, CDC or ADCC were not observed. Furthermore, the treatment with anti-CD47 in a mouse model resulted in the inhibition of MM cell growth, without affecting BM microenvironment [50].

Although the use of anti-CD47 mAbs promotes anti-MM activity alone, it is possible to implement the antitumor effect of this drug in combination with CD38 targeting agents, such as DARA [11,53]. Storti et al. showed that treatment with DARA increases the MM-killing and this effect has been correlated with the presence of a subset of CD14^+^ CD16^+^ monocytes. Furthermore, they observed that combination treatment DARA/anti-CD47 increases the killing of MM cells that survived only DARA treatment alone [11]. For the first time, the authors reported that the inhibition of CD47, “don′t eat me” antigen, enhances the anti-MM effect of DARA, shedding light on a future application of anti-CD47 mAbs in the therapeutic field.

Conversely, the potential role of CD47 blockade in combination with other therapies based on mAbs is still controversial [54,55,56]. Some authors reported that SLAMF7 expression is not required for CD47 blockage-induced phagocytosis in non-Hodgkin′s Lymphoma models, but others opined that its role in MM may be different [54]. It has been reported that the expression of SLAMF7 on macrophages is required for phagocytosis during treatment with anti-CD47 Abs [56]. Overall, the available data showed that CD47 could represent a promising new druggable target to treat MM.

### 4.3. Cyclophosphamide (Cy)

The anti-MM effect of DARA could be enhanced by Cy, in particular through an increased ADCP. Cy has been demonstrated to induce an acute secretory activating phenotype in tumor cells, that leads to macrophage infiltration and phagocytic activity in the BM [57]. Some authors reported that exposure of MM cell lines to low doses of Cy leads to a downregulation of CD47, which greatly increases macrophage-induced ADCP of DARA-coated MM cells [58]. Moreover, the results of the Phase 1B study NCT02955810, where DARA was used in combination with bortezomib, Cy, and dexamethasone (CyBorD DARA) as initial induction before autologous stem cell transplantation, were recently published [59]. In this study, they observed a significant reduction of CD47 on MM cells, and an increase of CD64 on BM macrophages, typical of an activated phenotype. Moreover, they reported a significant increase in the levels of tumor necrosis factor-alpha (TNF-α) and interferon-gamma (IFN-γ) in the patient’s serum, consistent with an antitumor response [59].

These findings suggest that MM cells may be more susceptible to ADCP after treatment with Cy and DARA together [59].

### 4.4. Histone Deacetylase Inhibitors (HDACi)

Panobinostat (PANO), is a pan-HDACi able to increase the expression of CD38 by PCs from MM patients [60]. Moreover, it has been demonstrated that the ADCC effect of DARA was increased by the pre-treatment of PANO [60]. Interestingly, PANO treatment did not affect CD38 expression on other cell types such as T cells [60].

Recently, Garcia-Guerrero et al. demonstrated that the specific inhibitor of the histone deacetylase 6 (HDAC6), ricolinostat, also increases CD38 RNA expression levels and CD38 molecules on the surface of MM cells. This upregulation is specific for MM cells and does not occur on T cells, leukemia, or lymphoma cells [61]. Moreover, the treatment with ricolinostat augments the ADCC by DARA against MM cell lines but not CDC effect [61].

Together, these in vitro evidence suggest that HDACi could be used in combination with DARA to potentiate the effect of the anti-CD38 mAb.

### 4.5. Urelumab: Anti-CD137 Monoclonal Antibody

NK cells acquire CD137 on their surface after having bound a Fc tail of an antibody with their CD16 receptor. Anti-CD137 mAbs, as urelumab, have been shown to enhance T cell mediate anti-tumor immunity and anti-tumor ADCC mediated by mAbs [62,63]. Ochoa et al. demonstrated that DARA-coated and ELO-coated MM cells induced the expression of CD137 on NK cell surface with an enhanced expression of CD25 and INF-γ production [64]. In a NOD scid gamma (NSG) mouse model reconstituted with human NK cells and MM cell lines, they have demonstrated that the treatment with urelumab in combination with DARA controls the tumor growth better than DARA alone treatment [64].

Moreover, phase I study combining low dose urelumab and ELO is ongoing (NCT02252263).

## 5. Therapeutic Strategies Combining Anti-SLAMF7 or Anti-CD38 mAbs and Immunomodulatory Drugs (ImiDs)

Several clinical trials evaluating the combination of anti-SLAMF7/anti-CD38 mAbs with ImiDs are currently ongoing in MM, with promising results. In this part of the review, we elucidate the rationale of these therapeutic strategies and provide a summary of mechanistic findings from pre-clinical studies in MM models.

### 5.1. ImiDs and Anti-SLAMF7 mAbs

Preliminary in vitro studies on the anti-SLAMF7 mAb, ELO, showed that the pre-treatment of either effector cells (peripheral blood mononuclear cells, PBMC) or target myeloma cells with clinically achievable doses of lenalidomide (LEN) enhanced ADCC-mediated lysis of MM cells triggered by ELO [26]. These results gave the rationale for the use of combined therapeutic strategies in MM patients. Data were confirmed by other groups using both in vitro and in vivo models of MM, which also added more details to the mechanisms behind this effect.

Specifically, Balasa et al. [65] demonstrated that ELO treatment increases NK cell recruitment to the tumor site and enhances ADCC, while LEN exerts direct cytotoxic effects against MM cells in a xenograft model [65]. On the other hand, the same group performed in vitro studies showing that the inhibition of MM cell growth after treatment with ELO and LEN was due to the modulation of NK cell function. Using a human peripheral blood lymphocytes (PBL)/myeloma co-culture model, the authors described the upregulation of IL-2Rα and CD54 (ICAM-1) expression on NK cells. Increased TNF-α levels and higher production of IL-2 by CD3^+^CD56^+^ lymphocytes in the presence of ELO+LEN compared with either agent alone was also described. TNF-α directly induced MM cell death, besides increasing NK cell activation; TNF- α neutralizing Abs reduced this effect [65]. The role of IL-2 in enhancing NK cytotoxicity and ADCC is also known [66], as well as the effect of LEN on IL-2 production by T cells [67], thus supporting the mechanist findings of this study.

More recently, Pazina et al. [68] described new trans-costimulatory signals following ELO engagement to SLAMF7. This interaction enhanced NK cell activation toward myeloma cells expressing ligands of NKp46, NKG2D, or potentially other ITAM-signaling receptors [69]. It is known that NKp46 and NKG2D are involved in NK-mediated cytotoxicity of myeloma cells [70]; however, their surface expression may be reduced on BM NK cells from MM patients [71]. Results from Pazina et al. [68] suggest that trans-costimulation by ELO enhances the signaling by these receptors, despite their reduced expression. More interestingly, the use of LEN, known to upregulate NKG2D ligand expression on myeloma cells [72,73], further increases their susceptibility to NK cell activity, thus potentially improving ELO therapeutic effect.

Overall, these studies thus explain the significant improvement of survival outcomes in RRMM patients treated with a combination of ELO and LEN.

### 5.2. ImiDs and Anti-CD38 mAbs

Similarly to ELO, the therapeutic combination of anti-CD38 mAbs with LEN or pomalidomide (POM) has shown encouraging clinical results in the treatment of RRMM patients, which are supported by preclinical data.

Van der Veer et al. [74] demonstrated the synergistic effect between DARA and LEN in the induction of ADCC cytotoxicity using an autologous system (BM mononuclear cells of MM patients), due to LEN activation of effector cells. Indeed, it is known that LEN stimulates NK cell proliferation and increases their production of IFN-γ, TNF-α, and granzyme B [67]. However, the authors did not report any effect of LEN on DARA CDC in these assays [74]. More interestingly, an up-regulation of DARA-dependent ADCC was described in PBMC isolated from MM patients during or just after LEN treatment, thus further supporting the potential benefits from this combination [74].

Other studies showed that LEN enhances in vitro DARA-induced myeloma cell lysis ADCC-mediated. This effect was mediated by an increased frequency of CD3^-^CD56^+^ NK cells, with no alterations of T-cell and monocyte compartments, even in patients’ refractory to LEN [75]. Ex-vivo experiments in humanized mice engrafted with MM cells from LEN refractory patients confirmed LEN ability to potentiate the DARA effect [75]. These results were in line with a study from Van der Veer et al. [76] where the synergism between DARA and LEN was more prominent in myeloma cells of patients refractory to LEN, [76] hence suggesting DARA potential to restore their susceptibility to these agents [75].

More recently, it has been suggested that vitamin D pathway activation can potentiate the synergism between LEN and MOR202-mediated ADCP [77] due to LEN ability to induce Cytochrome P450 Family 27 Subfamily B Member 1 (CYP27B1) expression in macrophages [12].

Another potential explanation for DARA-LEN synergism could be ImiDs ability to decrease the frequency of inhibitory T cell populations in vitro [78]. Indeed, a study by Feng et al. [16] demonstrated that LEN upregulates CD38 expression on T regs and increases the fraction of CD38-high T regs, consequently sensitizing this population to the anti-CD38, ISA [16].

In addition, a study by Bolzoni et al. [79] previously demonstrated that ImiDs in vitro treatment up-regulates CD38 expression on MM cells [79]. This finding was recently supported by Fedele et al. [80], which also described Ikaros and Aiolos degradation as a molecular mechanism behind this effect [80]. Moreover, the authors investigated the activity of DARA in combination with LEN and found a direct correlation between the additive effect of the treatment and the increased CD38 surface expression on MM cells [80]. On the other hand, no effect on CD38 expression was observed in NK cells, indicating that the synergistic effect on the effector cells is not mediated by this mechanism [80].

Lastly, Jiang et al. [19] showed that POM, more potently than LEN, enhances ISA both direct killing of MM cells, mediated by Caspase3/7 activation, and indirect cytotoxicity mediated by effector cells [19]. Moreover, POM/ ISA synergism is enhanced in CD38-high MM cells with mutated p53 [19]. These data, together with clinical POM activity against resistant MM with del (17p), support the use of POM/ISA combination even in high-risk patients.

These pre-clinical results thus give the rationale to combine ImiDs with anti-CD38 mAbs in the therapy against MM.

### 5.3. Sequence of Anti-SLAMF7 and Anti CD38 mAbs in RRMM

Up today, the optimal sequence for the administration of anti-SLAM7 and anti-CD38 mAbs in RRMM patients is unknown. DARA and ELO-based regimens have never been compared head-to head in RRMM patients. However, a retrospective clinical study showed that DARA retains its response rate whether administered as the first or second mAb used for the treatment of RRMM. On the other hand, the response rate of ELO seems to be reduced when ELO was administered after DARA [81]. Pre-clinical data may give some suggestions on the hypothesis that the previous use of anti-CD38 mAbs may reduce the efficacy of ELO. It is known that NK cells are reduced during DARA treatment retaining the ADCC capacity [82]. However, NK reduction levels could impact on the clinical effect of ELO mainly based on the binding and the activation of NK cells [83].

Moreover, in the ELOQUENT-2 clinical study it has been reported that the greatest PFS benefit in favor of ELO treatment was obtained in RRMM patients with a times of diagnosis longer or equal to a median of 3.5 years and those in relapse after a first line of treatment [84], suggesting that the efficacy of ELO in combination with ImiDs was greater in MM patients with a less refractory disease and with few line of previous treatments. However, recent clinical data (ELOQUENT-1) indicate that the addition of ELO to LEN and Dexamethasone did not improve the PFS, as compared to the treatment with LEN and Dexamethasone alone [ClinicalTrials.gov Identifier: NCT01335399].

Overall, these clinical evidences support the hypothesis that the use of ELO would precede the use of DARA in an optimal sequence of treatment in RRMM, whereas the role of ELO in newly diagnosed MM patients remains to be defined.

## 6. Anti-BCMA Targeting Antibodies

Along with CD38 and SLAMF/7, other possible therapeutic targets were identified in the last years to overcome MM cell drug resistance. B-cell Maturation Antigen (BCMA), also known as TNFRSF-17, is a TNF receptor (TNFR) family protein highly expressed by human MM cells [85]. BCMA has a critical role in the regulation of the survival of long-lived PCs, while it is not required in B cell differentiation as shown by the BCMA knock-out [86]. BCMA is selectively expressed by PCs but not by B lymphocytes, hematopoietic stem cells and other normal tissue cells [87,88]. This evidence makes BCMA a suitable target for the design of an immune-therapeutic approach. A panel of anti-BCMA mAbs have been developed showing cytotoxic activity in vitro against MM cells as either a naked IgG or a drug conjugate [89]. Moreover, innovative new generation anti-BCMA mAbs as antibody-drug conjugate and bispecific have been generated [90,91].

GSK2857916 is the first therapeutic anti-BCMA antibody-drug conjugate [92]. GSK2857916 is a humanized IgG1 anti-BCMA antibody conjugated with the toxin monomethyl auristatin F (MMAF), through a non-cleavable linker, with a high affinity for BCMA [92]. In a pre-clinical study, GSK2857916 induced MM cells killing [92]. In a xenograft mouse model the treatment was able to induce a complete MM regression [92].

GSK2857916 namely Belantamab Mafodotin was investigated in monotherapy in a phase I trial in RRMM patients [93,94]. The randomized phase II trial (DREAMM-2) [95], which enrolled 196 highly pre-treated MM patients in two cohorts with a different drug schedule. All MM patients enrolled are refractory to IMIDs and PIs, and previously treated with anti-CD38 mAbs. A recent update of the study, with a median follow-up of 9 months, indicates an overall response rate (ORR) of about 30% and showed a median PFS of 2.8 and 3.9 months in the two different cohorts, respectively; 1-year Overall Survival (OS) probability was 53%. Other studies with Belantamab Mafodotin either as single-agent or in combination with other drugs are currently ongoing. Moreover, many others anti-BCMA mAbs drug conjugate are in clinical development [96].

Another approach to target BCMA is based on bispecific antibodies (BsAbs), which deliver T cells against a target antigen through the induction of an immunological synapse [97]. BI 836909 is a bispecific T-cell engager (BiTE) designed as two linked single-chain variable fragments (scFvs) specific for BCMA and CD3ε at N-terminal and C-terminal, respectively [98]. Fase I-III clinical trial are ongoing in RRMM patients.

## 7. Conclusions

In conclusion, the introduction of mAbs have brought new immunotherapeutic approaches improving the clinical outcomes in the treatment of MM patients. However, many patients still relapse or do not respond to these agents. The unique mechanisms of action, low cytotoxicity, and safety profiles make mAbs an ideal constituent to be used in combination with current therapeutic regimens, which could improve the response rate and possibly overcome resistance.

**Figure 1:**
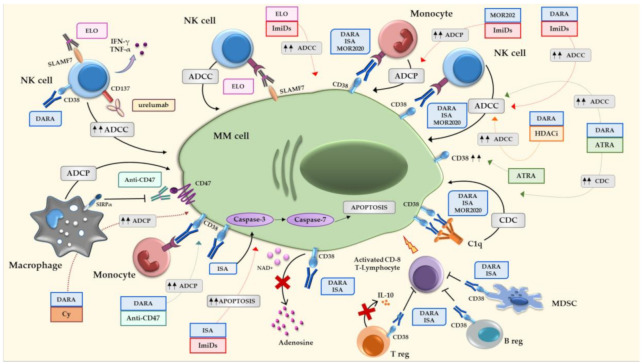
Mechanisms of actions of anti-CD38 and anti-SLAMF7 alone or in combination with other therapeutic agents. ADCC, antibody-dependent cell-mediated cytotoxicity; ADCP, antibody-dependent cellular phagocytosis; ATRA, all-trans retinoic acid; B reg, regulatory B cell; CD, complement-dependent cytotoxicity; Cy, cyclophosphamide; DARA, daratumumab; ELO, elotuzumab; HDACi, histone deacetylase inhibitors; IFN-γ, interferon-γ; ImiDs, immunomodulatory drugs; ISA, isatuximab; MDSC, myeloid-derived suppressor cells; MM, multiple myeloma; NAD, nicotinamide adenine dinucleotide; NK, natural killer; SLAMF7, signaling lymphocytic activation molecule family member 7; T reg, T regulatory cell; TNF-α, tumor necrosis factor–α; SIRPα, signal regulatory protein-α; IL, interleukin; C1q, complement component 1q.

Several mAbs able to target CD38 and SLAMF7, expressed by both MM cells and the immune microenvironment cells, have been developed. Combination therapy with IMiDs^®^ seems to potentiate the effect of the mAbs, anti-CD38 and anti-SLAMF7, compared with single-agent treatment, reaching significant clinical effects even in RRMM patients. Figure 1 summarizes the different mechanisms of actions of the anti-CD-38 and anti-SLAMF7 mAbs approved for the treatment of MM patients.

In this context, there is a need for novel strategies to improve MM cell killing by mAbs. Pre-clinical evidence indicates that different approaches may increase the efficacy of mAbs as the use of ATRA, Cy, or the combination with the anti-CD47 and anti-CD137 mAbs. These results gave the rationale for the ongoing clinical trials on these combination therapies, which could improve MM patient treatment (Figure 1).

New mAbs have been also developed to treat MM patients, refractory to several lines of treatment including those to anti-CD38 mAbs. Among these anti BCMA mAbs seems to be more promising.

To conclude, a better understanding of the mechanisms of action of mAbs will permit to develop novel approaches and drug combinations to improve the response rate of the mAbs and to overcome their resistance.

## Abbreviations

ADCC	antibody-dependent cell-mediated cytotoxicity
ADCP	antibody-dependent cellular phagocytosis
ATRA	all-trans retinoic acid
B reg	regulatory B cell
CDC	complement-dependent cytotoxicity
Cy	cyclophosphamide
DARA	daratumumab
ELO	elotuzumab
HDACi	histone deacetylase inhibitors
IFN-γ	interferon-γ
ImiDs	immunomodulatory drugs
ISA	isatuximab
MDSC	myeloid-derived suppressor cells
MM	multiple myeloma
NAD	nicotinamide adenine dinucleotide
NK	natural killer
SLAMF7	signaling lymphocytic activation molecule family member 7
T reg	T regulatory cell
TNF-α	tumor necrosis factor–α
